# Gender Differences in Lower Extremity Stiffness during a Single-Leg Landing Motion in Badminton

**DOI:** 10.3390/bioengineering10060631

**Published:** 2023-05-23

**Authors:** Yanan Zhang, Zhe Hu, Bairan Li, Xuan Qiu, Ming Li, Xiangwei Meng, Sukwon Kim, Youngsuk Kim

**Affiliations:** 1 Department of Physical Education, Jeonbuk National University, Jeonju 54896, Republic of Korea; 202055397@jbnu.ac.kr (Y.Z.); huzhe0710@jbnu.ac.kr (Z.H.); lming@jbnu.ac.kr (M.L.); mengx@jbnu.ac.kr (X.M.); 2 Department of Physical Education, Putian University, Putian 351100, China; libairan0526@ptu.edu.cn; 3 Department of Physical Education, Yichun University, Yichun 336000, China; 208144@jxycu.edu.cn

**Keywords:** badminton, single-leg landing, ACL injury, lower extremity stiffness

## Abstract

In general, at the same level of exercise, female athletes are three to six times more likely to injure an anterior cruciate ligament (ACL) than male athletes. Female athletes also had higher rates of ACL injury than males in a single-leg landing task after a backcourt backhand side overhead stroke in badminton. In many previous studies, stiffness of the musculoskeletal system in the lower limbs has been reported as a potential factor contributing to differences in ACL injury rates between genders. The purpose of this study was to describe the differences between genders in leg and knee stiffness in male and female athletes during a single-leg landing action after the backhand side overhead shot in the backcourt. Eight male athletes and eight female athletes participated in this test. Leg stiffness and knee stiffness were calculated separately for male and female athletes during the landing phase. The results showed that both absolute and normalized leg stiffness were lower in female athletes than in male athletes (*p* < 0.05). And both absolute and normalized knee stiffness were also lower than male athletes (*p* < 0.05). The low leg stiffness and knee stiffness demonstrated by females in this single-leg drop task compared to male athletes may indicate that females have lower dynamic leg stability than males during the drop, which may lead to hypermobility of the knee joint and may put females at a higher risk of injury in this high-risk maneuver for non-contact cruciate ligament injuries.

## 1. Introduction

Previous investigations have shown that anterior cruciate ligament (ACL) injuries may be the most severe lower limb injuries in badminton [1,2]. The most common type of injury among lower extremity ACL injuries is the non-contact ACL injury, accounting for seventy percent of all cases. This type of injury typically occurs more frequently in rapid sports that involve lateral cutting, twisting, and landing [3]. Competitive badminton is a complex sport involving high-frequency, high-intensity sudden stops, changes of direction, and landing. Furthermore, studies have indicated that female athletes typically experience ACL injury rates three to six times higher than their male counterparts at the same level of exercise [4]. Previous studies have shown that ACL injuries often occur when most or all of the body weight is transferred to one foot [5]. Through analysis of videos of badminton competitions, some academics have found that non-contact ACL injuries are most commonly suffered when a player lands on a single leg after performing a backhand side overhead stroke in the backcourt [6]. Additionally, epidemiological studies have indicated that in the sport of badminton, female athletes are at a higher risk of sustaining injuries during single-leg landing actions after executing backhand side overhead shots, compared to males. These injuries appear to occur most frequently during the landing phase [6,7].

Previous studies have identified both intrinsic and extrinsic risk factors for the occurrence of cruciate ligament injuries in different genders. Most authors have proposed various factors, including the influence of hormones and menstrual cycles, as well as anatomical, genetic, and neuromuscular differences between male and female athletes [8,9,10]. However, as the cause of ACL injury may be attributed to a complex interplay of risk factors, several studies have suggested that biomechanical factors may partially explain the gender difference in the incidence of ACL injury [11], and it is believed that stiffness of the musculoskeletal system is one of the biomechanical factors that may contribute to the sex differences in ACL injury rates [12].

The stiffness of the human body reflects its capacity to withstand the deformations caused by the ground reaction force (GRF) [13,14]. The spring-mass model reflects the characteristics of human jumping motion and is employed to approximate the musculoskeletal system of the lower extremity [15]. The term ‘leg stiffness’ is used to describe the resistance to change in the overall length of the leg following the application of an internal or external force. Leg stiffness can be controlled by adjusting the stiffness of individual joints. Joint stiffness is controlled by a variety of biomechanical factors, including muscle activation and strength [16,17,18], reflexes [19], antagonist co-activation, and lower limb kinematics during ground contact [20,21,22,23]. Joint stiffness can be controlled by altering the range of motion of the lower limb joints and the associated muscle activity [24]. Leg stiffness has been shown to be an important indicator of the risk of sports injury. Through numerous studies, an increasing amount of evidence indicates that both excessively high and excessively low stiffness can significantly contribute to a higher incidence of musculoskeletal injuries [7,12,25]. Previous studies have shown that excessive leg stiffness may increase peak forces and reduce the lower limb range of motion, the combination of which usually increases loading rates [26]. The combination of increased peak forces, loading rates, and impact forces can make people more susceptible to skeletal injuries [27]. Moreover, insufficient leg stiffness may lead to increased pressure on the passive supporting structures of the knee during the landing process, compromising joint stability and resulting in excessive joint motion. This can elevate the risk of non-contact soft tissue injuries, particularly knee ligament injuries [13].Women exhibit lower muscle stiffness than men when controlled open-chain measurements are performed on the isolated knee [25,28]. In addition, a prospective study found that bilateral differences in preseason leg stiffness increased the likelihood of non-contact lower limb injuries [29]. Some scholars have found that lower limb stiffness was generally lower in female than in male athletes when comparing male and female athletes performing jump landings and volleyball block jump landings. They suggest that the reduced stiffness in female athletes may result in reduced knee stability, causing excessive joint motion at the moment of landing, which may put female athletes at higher risk of ACL injury [30,31]. Therefore, by measuring the variables of lower limb leg stiffness and joint stiffness, we can gain more insight into the potential risk factors for multiple cruciate ligament injuries in female athletes than in male athletes during sports. Previous studies have investigated gender differences in lower extremity stiffness in some two-legged jumping events [32], however, no gender differences in lower limb stiffness were investigated during the movement of landing on one leg after a backhand overhead stroke in the backcourt of badminton, where female athletes are at higher risk of ACL injury (6 male and 15 female badminton players) [6]. To gain more insight into the possible factors that place female athletes at a higher risk of ACL injury than male athletes in single-leg landings after backcourt lateral backhand overhead strokes, a high-risk movement for ACL injury in badminton, and to provide injury prevention strategies for athletes, coaches, and rehabilitation practitioners, it is necessary to examine the gender bias in leg stiffness and knee stiffness during single-leg landings in both male and female athletes.

Therefore, we hypothesized that when badminton players landed on a single leg after a backhand overhead shot in the backcourt, female athletes may exhibit relatively less lower extremity stiffness compared with male athletes.

## 2. Materials and Methods

### 2.1. Subjects

In our study, we recruited 16 experienced badminton players, comprising 8 male and 8 female players (Table 1). All participants were members of the university’s badminton team and trained at least 6 times per week, with each training session lasting for 3 h. None of the participants had a history of surgery or injury to the trunk, spine, or extremities, limited range of motion in any joint, or muscle weakness, as confirmed by a review of their medical records. All subjects held the racket with their right hand. Prior to participating in the study, all subjects read and signed the informed consent form. This study received approval from the ethics committee of Jeonbuk University (JBNU2022-04-008-001).

### 2.2. Preparation for Testing

The three-dimensional motion data use a motion capture analysis system (OptiTrack, NaturalPoint, Inc., Corvallis, OR, USA) including 13 high-speed infrared cameras, and collects experimental data from each participating subject at a sampling frequency of 120 HZ. Each subject’s anatomical landmarks were equipped with 57 reflective markers. Ground reaction forces were collected by a force plate embedded in the floor (OR6-6-2000 force platform, AMTI Inc., Plano, TX, USA) at a sampling frequency of 1200 Hz. The motion analysis system and force plate were set up simultaneously before testing. After each test, the 3D motion data were transferred to Visual3D software (Professional 6.0, C-Motion Inc., Germantown, MD, USA) for data analysis of joint angles and other features.

### 2.3. Test Procedure

**Figure 1:**
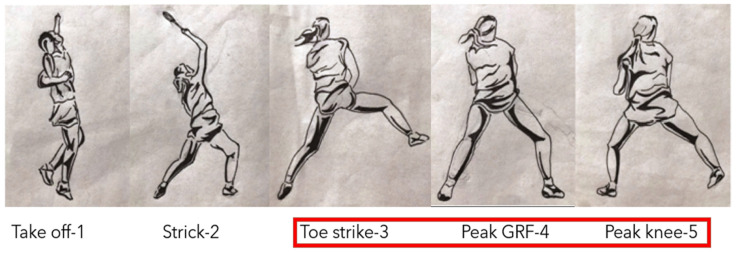
The single-leg landing motion after the backhand side overhead shot in the backcourt of badminton is divided into five stages: take off, strick, toe strike, peak GRF, and peak knee [5]. Red boxes indicate the landing stages analysed in this study.

We provided clear explanations of the test procedure and purpose to each subject prior to the commencement of the official test. Additionally, we recorded the subjects’ basic information, including gender, age, weight, height, and years of experience playing badminton (Table 1). Based on previous research, the actions described by Kimura in badminton with a higher risk of ACL injury were simulated (Figure 1) [5]. Each subject performed a task associated with a high-risk posture for ACL injury, which involved taking one step back to the backhand side of the badminton court to perform an overhead stroke followed by a single-leg landing. Considering that ACL injuries seem to frequently occur during the landing phase, we defined the landing phase in this study as the period from the initial contact point on the force plate to the point of maximum knee flexion during the testing [33].

**Figure 2:**
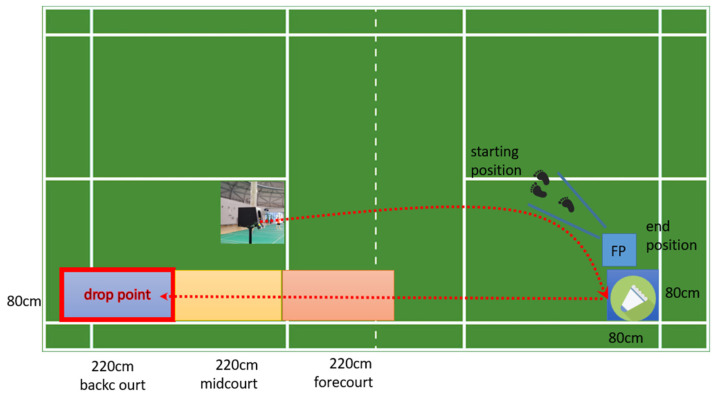
The shuttlecock service machine delivers the shuttlecock to the same location at the same speed and frequency. The subjects stepped back from the starting point to the backhand side of the badminton backcourt to perform the landing task. On landing, they dropped their left foot into the force plate and hit the badminton to the designated area. And quickly return to the starting position within 3 s of the end of the action. The red dotted lines show the sending line of the shuttlecock, and the flight path of the shuttlecock after it has been hit, respectively. The different boxes indicate where the shuttlecock will land and we require that the shuttlecock must land in the blue box after being struck to be recorded as a successful experiment.

To minimize individual differences and ensure the accuracy of our experimental results, we enlisted the help of an experienced trainer to demonstrate the proper test movements to all participants. To ensure consistency in every movement, we marked lines at a 45-degree angle on the badminton court and instructed each participant to land on the force measuring plate with one leg after hitting the backhand over the ball in the badminton backcourt. They were then required to quickly return to the starting point (see Figure 2). To reduce the risk of injury during the test, each participant was asked to perform a 15 min warm-up session consisting of jogging, jumping, and several high-clear swings prior to the formal test. Participants were then asked to perform three to five successful trials, with a 30 s rest period in between each test to avoid fatigue. To further minimize potential errors in the experiment, we provided each participant with the same badminton racket and shuttlecock, as well as the same experimental equipment.

### 2.4. Leg and Knee Stiffness Calculations

The spring-mass model consists of a body and linear leg springs supporting the body. Calculations of lower extremity leg stiffness and knee stiffness were performed using a spring-mass model as presented in previous studies [34]. Leg stiffness is defined as the ratio of the maximum ground reaction force (GRF) to the maximum leg pressure during the landing phase. The calculation formula is as follows:

Kleg (N/m) = peak GRF vertical/ΔL_COM_

where peak GRF is the maximum vertical ground reaction force. ΔLCOM is the vertical displacement of the center of mass (COM) from initial contact to maximum knee flexion. We will calculate the center of mass in Visual3D using a full-body biomechanical model based on the position and inertial properties of each body part. GRF data were normalized to body weight (BW), COM shifts were additionally normalized to height, and Kleg was normalized to weight and height. Both will be reported as absolute values and normalized values, respectively.

Joint stiffness was defined as the ratio of the joint moment changes to joint flexion during the landing phase. Knee joint stiffness is calculated using the formula:

Kjoint (Nm/rad) = ΔMjoint/Δθjoint

In this formula, ΔMjoint represents the change in joint moment between the moment of initial touchdown and the peak knee moment, and Δθjoint is the angular displacement of the knee joint between the moment of initial touchdown and the peak knee moment.

### 2.5. Data Processing and Analysis

All marker trajectory data were recorded and digitized using 3D motion capture system MotiveBody 2.2.0 software (OptiTrack, LEYARD, Buffalo Grove, IL, USA). Sagittal, frontal, and horizontal kinematic and kinetic data of the left hip, left knee, and left ankle were processed using Visual 3D Professional 6.0 software (C-Motion, Inc., Germantown, MD, USA) and presented as mean and standard deviation (SD) of the test. The impact phase of landing was analyzed first, as it is the critical period for non-contact ACL injuries, which commonly occur during the early stages of landing [35]. In this study, the landing phase was defined as the period from initial contact with the force plate to the point of maximum knee flexion in the test, considering that ACL injuries usually occur during this phase [33]. The angular position of the knee is defined as the calf relative to the thigh and using X (flexion/extension), Y (adduction/abduction), and Z (internal rotation/external rotation), using the right-hand convention. Directions are fixed by sign, being positive for flexion, negative for extension, positive for adduction, negative for abduction, positive for internal rotation, and negative for external rotation. Joint torque is calculated using the inverse dynamics method by combining force plate data with kinematic data. We analyzed the data collected in the experiment using SPSS19 software. After calculating the data collected from badminton players, the calculated kinematic and kinetic data were compared statistically using independent *t*-tests. *p* < 0.05 was considered statistically significant.

Absolute leg stiffness and leg stiffness normalized to height and weight, as well as absolute knee stiffness and knee stiffness normalized to height and weight, were separately calculated for each subject, including eight male athletes and eight female athletes. Before conducting an independent sample *t*-test, normality analysis and chi-square tests were performed on the data of the male and female athletes. The two sets of data are from normally distributed populations with flush variance and satisfy independence. Subsequently, independent sample *t*-tests were performed on the data from the male and female athletes to examine gender differences between initial ground contact and maximum knee flexion. The tests included changes in vertical GRF, vertical COM displacement, leg stiffness, knee moment change, knee flexion angle displacement, and knee stiffness.

## 3. Results

### 3.1. Leg Stiffness

In Table 2, we observe that the absolute change GRF is significantly higher in male athletes compared to female athletes (*p* < 0.05). There is no significant difference in the vertical displacement of the center of gravity between male and female athletes. However, the calculated absolute leg stiffness in men is significantly higher than that in women, and this difference is statistically significant (*p* < 0.05).

From Table 3, we can see that the normalized variation in GRF was also significantly greater in males than in female athletes (*p* < 0.05). There was no significant difference between male and female athletes in terms of normalized vertical displacement of the COM. However, there was also a significant difference in normalized leg stiffness between male and female athletes (*p* < 0.05).

### 3.2. Knee Joint Stiffness

In Table 4, we can see that there is a significant difference in the absolute change in knee torque between male and female athletes (*p* < 0.05). In addition, the absolute change in knee flexion angle was significantly smaller in male athletes compared to female athletes, a significant difference (*p* < 0.05). Additionally, the absolute knee stiffness calculated from these measurements was significantly greater in male athletes than in female athletes, a significant difference (*p* < 0.05).

In Table 5, we can see that there is no difference between male and female athletes in the amount of change in knee moment after normalization, but there is a significant difference between male and female athletes in the calculated normalized knee stiffness (*p* < 0.05). Female athletes had significantly lower knee stiffness than male athletes and there was a significant difference (*p* < 0.05).

## 4. Discussion

This study aimed to investigate the differences between genders in regard to leg and knee stiffness among male and female athletes during single-leg landing after a backhand overhead shot in the backcourt of badminton. The results of this study support our hypothesis, as we found that female athletes exhibited lower leg and knee stiffness in this landing task compared to males. This could be a potential factor contributing to the higher risk of non-contact ACL injuries among female athletes in this type of landing task compared to males.

In our study, we observed that during a single-leg landing after a backhand overhead shot in the backcourt of badminton, male athletes exhibited significantly higher absolute leg stiffness (16.95 KN/m ± 4.17) and normalized leg stiffness (42.56 BW/ht ± 10.66) compared to female athletes, who had lower absolute leg stiffness (9.45 KN/m ± 1.98) and normalized leg stiffness (26.07 BW/ht ± 4.72). Similar results have been seen in previous studies on two-legged landing tasks. Ward, Rachel E et al. compared the stiffness of male and female dancers and male and female athletes during jumping and landing tasks and they also found that female athletes had less leg stiffness than male athletes [33]. A previous study by Padua et al. also found that, compared to males, female athletes had significantly less stiffness in the lower limbs during jumping compared to males [12]. However, the mean leg stiffness values for the male and female athletes in this study were higher than in previous studies, which may be due to differences in our single-leg landing task.

In the single-leg landing task, we investigated and we observed that males had a larger absolute vertical GRF variation compared to females. This difference may be attributed to the fact that males generally have significantly higher body weight. However, even after normalizing the data based on weight, males still exhibited a higher vertical GRF compared to females. Additionally, there were no significant differences in both absolute and normalized vertical COM displacement between males and females. However, when examining both absolute and normalized leg stiffness, calculated based on these variables, we found that female athletes had significantly lower values than male athletes. When performing high-intensity functional tasks, the stiffness of muscles and joints plays a critical role in the stability of the functional joint [36]. Stiffer muscles can more quickly and effectively resist sudden joint displacement, possibly as a protective mechanism to prevent acute knee joint injuries [13]. In addition, the passive and dynamic resistance to joint displacement of larger muscles is associated with a greater muscle cross-sectional area [37,38].In the same task, female athletes exhibit lower leg stiffness compared to male athletes, which may indicate a weaker ability for females to resist external compressive and resistance forces during functional single-leg landing tasks, leading to lower dynamic stability of the legs than males. As dynamic leg stability can be reflected in leg stiffness, stiffness is interpreted as the ability of the leg to resist compressive forces from external forces, such as flexion of the hip, knee, or ankle during landing [31]. And this may be related to anatomical differences between males and females, as males typically have greater muscle mass than females. And exhibiting low leg stiffness may increase the stress on the passive support structures of the knee during landings in women, leading to hypermobility of the joint and thus increasing the likelihood of non-contact cruciate ligament injuries in female athletes [31]. However, Granata, Padua, and Wils in their study [12] it is not clear whether the cause of the gender difference in leg stiffness is due to the physical characteristics of male and female athletes. However, in our results, the male athletes still had significantly higher leg stiffness than female athletes even after normalizing for weight and height.

In the single-leg landing motion following a backhand overhead shot in the backcourt of badminton, female athletes showed a significant difference in absolute knee joint stiffness (2.57 N.m/° ± 1.099) compared to male athletes (5.17 N.m/° ± 0.789). Additionally, the normalized knee joint stiffness of female athletes (0.0026 BW.ht/° ± 0.0001) was significantly lower than that of male athletes (0.0041 BW.ht/° ± 0.0008). This finding supports our hypothesis that in this single-leg landing task, female athletes exhibit lower leg stiffness and knee joint stiffness compared to male athletes. These findings in our study align with similar results observed in another investigation on volleyball blocking jumps [31]. These results suggest that lower knee joint stiffness may be a potential risk factor for ACL injury in female athletes during single-leg landings. In this study, the decrease in normalized knee joint stiffness among female athletes compared to males may be attributed to their less leg stiffness (absolute and normalized values). Joint stiffness (Kjoint) is a fundamental measure for all lower extremity tasks, as the stiffness of joint components within the system ultimately affects the overall system stiffness (i.e., Kvert or Kleg) [39,40,41]. Joint stiffness is influenced by various factors, including the stiffness of each tendon unit that crosses the joint. The stiffness of muscle-tendon units, in turn, depends on both muscle stiffness and tendon stiffness [20]. It is important to note that muscle stiffness is determined by the level of muscle activation; in other words, muscle stiffness can be indicated by the degree of muscle activation [18,42]. During high-intensity lower extremity exercise, the combined contraction of the hamstrings and quadriceps increases joint contact force, limits intra-articular motion (thus keeping the joint stable), and provides protection to the knee joint against movements that carry a high risk of injury, such as knee abduction and twisting movements [43]. During high-load functional tasks, the greater the resistance of the muscles to knee joint rotation, the higher the likelihood of maintaining dynamic stability and increased stiffness in the knee joint. Consequently, there is a reduced likelihood of stressing passive structures, such as the cruciate ligaments. Therefore, knee joint stiffness may be a significant factor in preventing cruciate ligament injuries. In our study, the lower knee joint stiffness observed in female athletes may be associated with insufficient activation of the knee joint antagonist muscles. Co-activation of the lower limb antagonist muscles is part of a strategy that can increase muscle stiffness and stability [44,45]. Previous studies have shown that during jump landing tasks, females attempt to partially alter lower limb stiffness by recruiting more quadriceps, and while large quadriceps activation may be an effective mechanism for modulating lower limb stiffness during jumping, it may have a detrimental effect on knee joint stability [32]. In addition, a previous study had found that an unbalanced recruitment strategy of the quadriceps and hamstrings was used more significantly in females compared to males during the landing task [32], previous studies have shown that when the hamstrings are stiffer it may be able to limit tibial anterior translation and shear forces and can relatively reduce the external forces exerted on the cruciate ligament [37].

In this single-leg landing task, we observed a significant difference in the knee flexion angle displacement between female athletes (36.97° ± 7.38) and males (28.88° ± 4.92). This disparity may be attributed to weaker hamstring strength and lower muscle activation, which limits the ability of muscle contraction to protect the ligaments, leading to reduced joint stiffness and lack of stability upon landing. Consequently, female athletes face a higher risk of cruciate ligament injury compared to their male counterparts during landing. Notably, our study also found significantly lower normalized knee stiffness in females compared to males, contradicting some conflicting results in the published literature [32]. This may also be caused by the fact that our present study was a single-leg landing task, unlike the previous two-leg landing task. Previous research showed that when knee stiffness was normalized for body weight, the differences between male and female athletes were not evident, indicating that the increased net external knee flexion moment after normalization could maintain similar normalized knee stiffness in female athletes compared to males [46,47]. Moreover, other studies have demonstrated that humans can autonomously control joint stiffness through muscle recruitment, thereby enhancing dynamic joint stability by adjusting the joint stiffness [48]. As previously mentioned, female athletes who used these strategies while jumping showed a more dominant recruitment pattern for the quadriceps compared to male athletes [32]. A lower knee flexion moment in female athletes may contribute to lower extremity stiffness and higher injury rates due to the limited ability of the quadriceps to balance [49].

When interpreting the results of this study, it is important to acknowledge and consider certain limitations. Firstly, the average age of the athletes we recruited was 20 years, which is probably younger than the age used in previous studies. As leg stiffness is influenced by the combined effects of hip, knee, and ankle stiffness [12], we focused solely on knee stiffness in this study. Furthermore, torsional joint stiffness is regulated by various biomechanical factors, including muscle activation and strength [16,17,18], reflexes [19], antagonist co-activation, and lower limb kinematics during ground contact [20,21,22,23]. In this study, we solely examined lower limb kinematic and kinetic data from male and female athletes during ground contact. However, it is important to supplement these findings with lower limb muscle surface electromyography to assess muscle strength and function during exercise tasks [50,51]. Additionally, future investigations could explore different muscle activation and exercise strategies to modulate lower limb stiffness under functional loading conditions.

## 5. Conclusions

In the single-leg landing motion, after a backhand overhead shot in the backcourt of badminton, compared with male athletes, female athletes showed significantly less absolute and normalized leg stiffness and less absolute and normalized knee stiffness during landing. This suggests that female athletes may be more susceptible to factors than males to non-contact ACL injuries during landing motion, as female athletes exhibit lower leg low dynamic stability and high knee hypermobility during landing than male athletes. In future training, female athletes should appropriately increase the stiffness of their lower limbs. However, further research is needed to determine the “optimal” stiffness level required to achieve functional tasks.

## Figures and Tables

**Table 1:** Basic information of male and female athletes participating in the experiment.

	Males (*n* = 8)	Females (*n* = 8)
Age (years)	21.5 ± 2.57	20.38 ± 2.19
Height (cm)	178.5 ± 2.21	167.6 ± 5.76
Body weight (Kg)	70.63 ± 6.17	61.38 ± 6.98
Experiences (years)	9.62 ± 1.95	10.75 ± 2.87

**Table 2:** Absolute change in vertical GRF, absolute vertical COM displacement, absolute leg stiffness (mean ± SD).

	Males	Females	*p* Value
Change in vertical GRF (N)	2339.16 ± 549.50	1235.44 ± 316.87	0.0002 *
Vertical COM displacement (m)	0.141 ± 0.025	0.131 ± 0.024	0.3852
Leg stiffness (KN/m)	16.95 ± 4.17	9.45 ± 1.98	0.0004 *

“*” indicates a significant difference *p* < 0.05.

**Table 3:** Normalized change in vertical GRF, normalized vertical COM displacement, normalized leg stiffness (mean ± SD).

	Males	Females	*p* Value
Change in vertical GRF (BW)	3.29 ± 0.73	2.03 ± 0.38	0.0006 *
Vertical COM displacement (ht)	0.079 ± 0.014	0.078 ± 0.013	0.9954
Leg stiffness (KN/m)	16.95 ± 4.17	9.45 ± 1.98	0.0004 *

“*” indicates a significant difference *p* < 0.05.

**Table 4:** Absolute change in knee joint moment, absolute knee flexion displacement, absolute knee stiffness (mean ± SD).

	Males	Females	*p* Value
Change in knee joint moment (N.m)	158.46 ± 32.31	106.67 ± 51.57	0.0304 *
Knee flexion displacement (°)	30.511 ± 3.442	40.989 ± 6.770	0.0015 *
Knee stiffness (N.m/°)	5.179 ± 0.789	2.57 ± 1.099	0.0002 *

“*” indicates a significant difference *p* < 0.05.

**Table 5:** Normalized change in knee joint moment, normalized knee flexion displacement, normalized knee stiffness (mean ± SD).

	Males	Females	*p* Value
Change in knee joint moment (BW.ht)	0.125 ± 0.022	0.104 ± 0.045	0.2713
Knee flexion displacement (°)	30.511 ± 3.442	40.988 ± 6.769	0.0015 *
Knee stiffness (BW.ht/°)	0.0041 ± 0.0008	0.0026 ± 0.001	0.0034 *

“*” indicates a significant difference *p* < 0.05.

## Data Availability

The data presented in this study are available on request from the corresponding author.
